# Enhanced interleukin-8 production in mononuclear cells in severe pediatric obstructive sleep apnea

**DOI:** 10.1186/s13223-019-0338-1

**Published:** 2019-04-16

**Authors:** Danbing Ke, Yuji Kitamura, Duncan Lejtenyi, Bruce Mazer, Robert T. Brouillette, Karen Brown

**Affiliations:** 1 Research Institute, McGill University Health Center, 1001 Decarie Boulevard, Montreal, QC H4A 3J1 Canada; 2 Department of Anesthesia, McGill University Health Center, 1001 Decarie Boulevard, Montreal, QC H4A 3J1 Canada; 3 Division of Pediatric Allergy and Immunology, McGill University Health Center, 1001 Decarie Boulevard, Montreal, QC H4A 3J1 Canada; 4 Department of Pediatrics, McGill University Health Center, 1001 Decarie Boulevard, Montreal, QC H4A 3J1 Canada; 5 Department of Anesthesia, McGill University Health Center, Montreal Children’s Hospital, 1001 Decarie Boulevard, Room B 04.2422, Montreal, QC H4A 3J1 Canada

**Keywords:** Children, Obstructive sleep apnea, Interleukin-8, Chronic intermittent hypoxemia

## Abstract

**Background:**

Obstructive sleep apnea (OSA) is a risk factor for cardiovascular disease, metabolic disorders, and cognitive dysfunction. Current thinking links chronic intermittent hypoxia (CIH) with oxidative stress and systemic inflammation. However, the sequence of events leading to the morbidities associated with OSA is poorly understood in children. Monocytes are known to be altered by chronic hypoxia. Thus in this prospective study, we investigated inflammatory cytokine profiles from cultures of peripheral blood mononuclear cells (PBMC) obtained from children with severe OSA and sleep-related CIH.

**Methods:**

Ten children with OSA (cases) and 5 age-matched children without OSA (controls) were recruited for study. Samples of plasma and PBMC were obtained before and after adenotonsillectomy. The levels of the inflammatory cytokines, interleukin (IL)-1β, IL-6, IL-8, IL-10, IL-12p70, and tumor necrosis factor-α (TNFα), were measured in both plasma and ex vivo culture supernatants of PBMC incubated with lipopolysaccharide (LPS) using the cytometric bead assay.

**Results:**

Upon activation of PBMC by LPS, the levels of IL-8 in the culture supernatants from cases were threefold higher than in controls. The levels of the other cytokines including IL-1β, IL-6, and TNFα, in culture supernatant of PBMC from cases showed no difference from controls; nor were there significant differences in plasma cytokine levels.

**Conclusion:**

We speculate that in young children with sleep-related CIH, an enhanced production capacity of IL-8 precedes the development of systemic inflammatory markers. Future work should evaluate IL-8 production capacity as a potential biomarker for OSA severity.

## Introduction

Severe obstructive sleep apnea (OSA) is characterized by repetitive episodes of disrupted breathing during sleep and recurrent decreases in blood oxygen saturation [1]. Untreated, severe OSA has important sequelae that negatively impact the well being and school performance of children [2–4]. The most common cause of severe pediatric OSA is adenotonsillar hypertrophy and adenotonsillectomy (T&A) remains the recommended first line treatment [5]. The indication for T&A in milder forms of OSA is controversial as the surgery itself is associated with both morbidity and mortality [6, 7]. A reliable biomarker for OSA severity could inform clinical decisions.

A hallmark of severe OSA is sleep-related chronic intermittent hypoxia (CIH). CIH can lead to systemic inflammation in animal models and in patients with OSA [8–10]. Indeed, in both adult and pediatric patients, an upregulation of prototypic inflammatory cytokines such as tumor necrosis factor-α (TNFα) and interleukin-6 (IL-6), arising from activation of the nuclear factor κB (NFκB) pathway, is reported in OSA [11]. The notion that OSA is a low grade chronic inflammatory disease has been suggested [12].

Clinical studies have focused on the association between plasma/serum biomarkers and OSA severity or outcomes [11, 13]. The role of cellular alteration(s) in severe OSA has been less explored, although cells such as monocytes are known to be susceptible to hypoxia [14, 15]. Thus, we assessed the production of classic inflammatory cytokines in ex vivo cultures of isolated peripheral blood mononuclear cells (PBMC) obtained from children with severe OSA who exhibited sleep-related CIH. We employed a sensitive cytometric bead array to simultaneously detect six inflammatory mediators, namely TNFα, IL-6, IL-8, IL-10, IL-1β and IL-12p70 before and following T&A.

## Materials and methods

### Subjects

The study was approved by the Research Institute of the McGill University Health Centre/Montreal Children’s Hospital (Study #13-427-PED) and was conducted in accordance with Good Clinical Practice Guidelines and Standard Operating Procedures. Informed parental consent was obtained as well as verbal assent, when applicable, from the child for blood procurement. A convenience sample of 10 children undergoing T&A (cases) and 5 age-matched controls (controls) was recruited between September 2014 and December 2015.

We recruited children with clinical and laboratory findings consistent with severe OSA. Inclusion criteria for cases were (1) symptoms of OSA and sleep-related CIH, and (2) an elective T&A performed before noon. Inclusion criteria for the controls were (1) an elective insertion of pressure equalizing tubes for chronic otitis media, (2) a negative history for symptoms of OSA, and (3) surgery scheduled before noon.

Exclusion criteria included acute tonsillitis, a history of cardiorespiratory, neurologic, craniofacial, immunodeficiency, or genetic disorders, a recent course of systemic steroids, daily inhaled or nasal corticosteroids.

Blood procurement in cases and controls was performed immediately following induction of anesthesia. It is our practice to re-evaluate the sleep related breathing in children with severe OSA following T&A. At this follow-up visit blood procurement was performed in the McGill University Health Center (MUHC) Pediatric Center for Innovative Medicine. Controls were not re-evaluated following surgery. Blood samples were sent to the central laboratory for the measurement of serum levels for C-reactive protein (CRP), erythropoietin, and complete blood count (CBC).

Clinical data were collected for age, weight, height, and gender. Body mass index was calculated as weight in kilograms divided by height in meters squared. Evaluation of OSA severity included the administration of a sleep disordered breathing quality of life questionnaire (SDBQoL). A nocturnal pulse oximetry study was also performed and used to determine the number of 4% dips in saturation from baseline, the nadir saturation (nSAT), and the McGill Oximetry Score (MOS). The MOS classified the severity of CIH according to the frequency and depth of desaturation events. Both the MOS3 and MOS4 classifications, having recurrent drops in hemoglobin saturation to < 80% and < 85%, respectively, were consistent with severe sleep-related CIH and a diagnosis of severe OSA [16].

### Isolation and stimulation of peripheral blood mononuclear cells (PBMC)

PBMC were isolated from heparinized whole blood by density gradient centrifugation (Ficoll-Paque PLUS, GE Healthcare Bio-science AB, Uppsala, Sweden) within 4 h following blood collection. Two mL of plasma samples were obtained and stored in microcentrifuge tubes at − 80 °C. The PBMC were stimulated in vitro with lipopolysaccharides (LPS, 10 µg/mL, Sigma-Aldrich, Oakville, Ontario, Canada) at 37 °C in the presence of normoxia and 5% CO_2_ for 24 h. Culture supernatants were collected by centrifugation and stored at − 80 °C prior to use. Unstimulated PBMC were used to determine baseline cytokine production.

### Determination of cytokine production using cytometric bead array

Cytokine levels in plasma and culture supernatants were determined by flow cytometry using the Human Inflammatory Cytometric Bead Array kit (BD Biosciences, Mississauga, Ontario, Canada). The fluorescence associated with the antibody-coated beads was captured using a BD FACS Canto-II flow cytometer (BD Biosciences) according to the manufacturer’s instruction and data analyzed using FCAP Array software v3.0 (BD Biosciences). The kit allows for simultaneous quantification of 6 inflammatory cytokines (IL-1β, IL-6, IL-8, IL-12p70, IL-10, and TNFα) with sensitivity comparable to that of conventional ELISA. If the readout reached the upper limits of detection, additional dilutions were performed and the assay repeated. If the readout was below the detection limit, the value was arbitrarily set to half the lower detection limit.

### Statistical analysis

Data analysis was performed with Graphpad Prism version 5.0 for Windows, (Graphpad Software, La Jolla California USA, http://www.graphpad.com). Clinical data were described with the mean ± standard error. Assay data were described with the median, minimum, and maximum. Differences between the case and control groups were assessed with Student’s t test for clinical data; Mann–Whitney U tests for cytokine levels. Differences before and after T&A were assessed with Wilcoxon paired test. A *p* value < 0.05 defined statistical significance.

## Results

### Cases

The reason(s) given for seeking medical attention were snoring ± frequent nocturnal awakenings ± witnessed apnea ± difficulty breathing during sleep. In addition to the above, two parents reported recurrent tonsillitis. The SDBQoL score ranged from 49 to 103, indicating a moderate to large impact of daily life. Tonsillar enlargement ranged from 2+ to 4+. Before T&A, 8 cases had MOS4; 2 had MOS3. The interval between T&A and follow-up was 82 days (minimum 49, maximum 142). One case did not return for follow-up; two additional cases declined verbal assent for blood procurement. Parents reported an improvement in symptoms. Re-evaluation of sleep disordered breathing was performed in 5 cases. The SDBQoL scores ranged from 21 to 38, indicating a small impact on daily life and the overnight pulse oximetry result was MOS1.

### Controls

The reason(s) given for seeking medical attention were recurrent otitis media and/or hearing loss.

Controls were older and heavier than cases. The CRP levels were within the normal range except for one case (46.7 mg/L) and one control (6.8 mg/L). The leukocyte counts and differentials showed no differences between cases and controls (Table 1).

**Table 1:** Demographic and clinical laboratory data of cases and controls

Variable	Cases (n = 10)	Controls (n = 5)	p value
Age (years)	2.7 ± 0.4	4.0 ± 0.6	0.07
Weight (kg)	13.1 ± 0.5	19.4 ± 2.1	*0.001*
BMI (kg/m^2^)	15.4 ± 0.6	16.5 ± 1.0	0.37
Gender (boys)	9	5	na
Ethnicity			
Caucasian	5	4	na
Black	5	1	
nSAT (%)	72.6 ± 1.5	91.3 ± 2.3	*<* *0.0001*
DI4 (events per hour)	26.9 ± 14.2 (n = 9)	1.5 ± 2.1 (n = 3)	na
Clinical lab testing			
CRP mg/L (normal range 0.0–5.0)	5.7 ± 4.6	1.8 ± 1.3	0.56
EPO mIU/mL (normal range 2.6–18.5)	8.1 ± 1.3	na	na
Hemoglobin g/L (normal range 105–135)	114.5 ± 2.7	124.2 ± 1.7	*0.03*
Leukocyte counts and differentials			
White blood cells (× 10^6^)	10.5 ± 1.2	9.5 ± 0.4	0.53
Polymorphonuclear cells (%)	32.2 ± 3.3	39.8 ± 4.5	0.20
Lymphocytes (%)	54.6 ± 3.2	49.4 ± 4.5	0.36
Monocytes (%)	10.1 ± 0.7	8.2 ± 0.5	0.09
Cytokine production capacity in PBMC stimulated with LPS (pg/mL) median (min, max)			
IL-1β	1360 (346; 3510)	4220 (1660; 5630)	0.08
IL-6	3190 (946; 18,000)	7000 (3340; 12,100)	0.13
IL-8	39,000 (11,000; 266,000)	12,100 (6330; 32,600)	*0.03*
IL-10	47 (2; 358)	304 (100; 336)	0.11
IL-12p70	nd	nd	na
TNFα	277 (124; 745)	444 (183; 734)	0.21

Italics values indicate* p* < 0.05

*CRP* C-reactive protein, *DI4* desaturation index 4%, *EPO* erythropoietin, *IL* interleukin, *LPS* lipopolyssacharide, *PBMC* peripheral blood mononuclear cells, *na* not applicable, *nd* not detected, *nSAT* nadir saturation, *TNFα* tumor necrosis factor alpha

### Plasma cytokine levels

Preoperative plasma inflammatory cytokines were evaluated in 8 cases. At follow-up samples were obtained in only three. All measured levels of inflammatory cytokines were low, both before (n = 8) and after (n = 3) T&A. Plasma cytokine levels were not measured in controls.

### IL-8 production by LPS stimulated PBMC was augmented in children with OSA

**Fig. 1:**
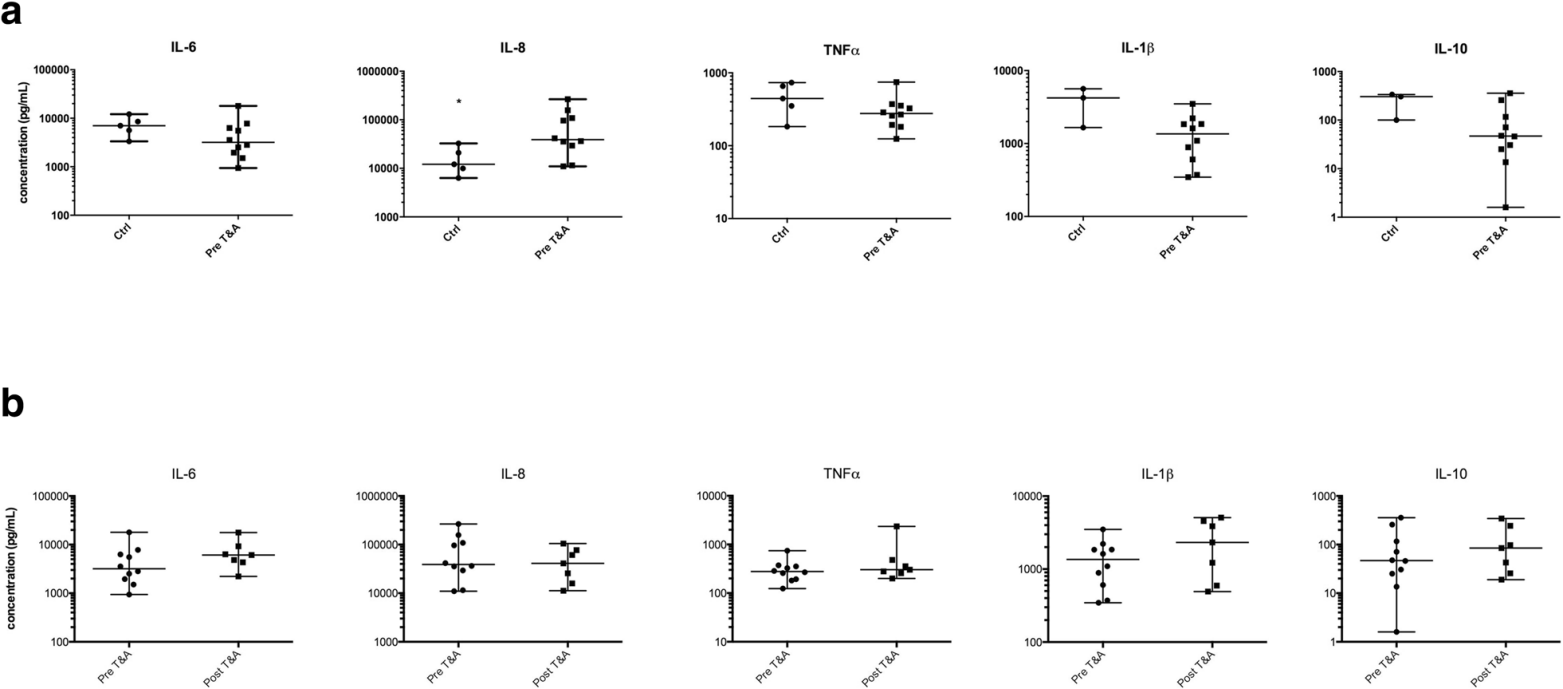
Median cytokine production capacity (pg/mL) after 24 h of incubation with lipopolysaccharide (LPS) in ex vivo cultures of isolated peripheral blood mononuclear cells in cases and controls. **a** Compares cases (n = 10) and controls (n = 5). **b** Compares cases (n = 7) pre and post adenotonsillectomy (T&A). Error bars represent the minimum and maximum values. *IL* interleukin, *TNFα* tumor necrosis factor alpha. *p < 0.05

Upon LPS stimulation, the concentrations of cytokines in PBMC culture supernatants were increased compared to unstimulated PBMC cultures; with the exception of IL-12p70. The extent of this increase varied. The stimulated IL-8 levels were threefold higher in cases (39,000 pg/mL) than controls (12,100 pg/mL) (p < 0.028) (Table 1) and IL-8 remained elevated at follow-up (Fig. 1a). In contrast, the LPS stimulated cytokine production capacity for IL-1β, IL-6, IL-10, and TNFα tended to be lower in cases compared with controls; differences were not statistically different. At follow-up cytokine production capacity for IL-1β, IL-6, IL-10, and TNFα tended to increase (Fig. 1b).

## Discussion

Children with a high MOS have severe OSA as quantified by polysomnographic criteria: the apnea–hypopnea index (AHI), the desaturation index (DI4), the respiratory arousal index (RAI), and the nSAT. A MOS3 correlates with a 95% confidence interval for AHI of 8.5 to 18.1; DI4 of 7.8 to15.1; RAI of 5.2 to 12.5; and nSAT of 77.7 to 84.2%. A MOS4 correlates with a 95% confidence interval for AHI of 26.5 to 53.3; DI4 of 26.3 to 53.7; RAI of 12.8 to 31.3; and nSATof 54.3 to 64.1% [16]. As DI4 is highly correlated with AHI [17], the values reported for cases in Table 1 as well as the clinical evaluation support a diagnosis of severe OSA. The major finding in this study was the selective elevation in the production capacity of IL-8 in children with sleep-related CIH, compared with controls.

Systemic inflammation has been linked to the development of cardiovascular, metabolic and neurocognitive sequalae of OSA [4, 10]. Increases in IL-6, TNFα and CRP, and a decrease in IL-10 in children with OSA are reported; all levels normalizing following T&A [10, 13, 18, 19]. A systematic review of 51 adult studies reported higher serum levels of proinflammatory markers in patients with OSA [11]. Both findings support the notion that OSA promotes a low intensity inflammatory state [12].

Although the cases exhibited disturbed sleep and sleep-related CIH, consistent with a diagnosis of severe OSA, the plasma levels of inflammatory cytokines were low as were the levels of TNFα and IL-1β, in PBMC cultured without LPS stimulation. Thus, there was no evidence of a systemic inflammatory response in these young children. In contrast Gozal et al. [10] reported a systemic inflammatory response in children with OSA. However the children in our study were younger (2.7 versus 6.5 years), had higher obstructive apnea indices (DI4 of 26.9 versus AHI of 13.3) and lower nadir saturations (72.6% versus 77.9%). Both age and the severity of sleep-related CIH are potential factors influencing IL-8 production capacity. We were not able to evaluate them as the small sample size precluded an adjusted statistical analysis. Their potential effects were mitigated by restricting the evaluation to young children with severe sleep-related CIH.

The finding that PBMCs stimulated with LPS for 24 h had a selective threefold increase in IL-8 production capacity in cases suggests a persistent, change in mononuclear cell function that was independent of activation of the NFκB pathway. IL-8 is a chemokine that plays a critical role in the host immune response and also in angiogenesis [20]. Metinko et al. reported that under conditions of anoxic preconditioning and oxidative stress IL-8 production in monocytes increased [15]. In the current study, although cultured under normoxic conditions, the PBMC samples were obtained from children exhibiting severe sleep-related CIH. Thus the circulating mononuclear cells were preconditioned with CIH. We speculate that the increased IL-8 production capacity was influenced by this stimulus; although the molecular pathways linking sleep-related CIH and IL-8 production remain to be elucidated. Alternate explanations for the increased IL-8 production capacity are also possible, such as the stimuli leading to tonsillar enlargement.

## Conclusion

The profiles of proinflammatory markers from cultures of isolated PBMC may represent an early event in the development of the systemic response to severe OSA and sleep-related CIH. Such profiles have been little studied in young children. A better understanding of the pathophysiology leading to the development of OSA would facilitate the identification of clinically relevant biomarkers for OSA severity in children.

## Abbreviations

CIH: chronic intermittent hypoxia
CRP: C-reactive protein
IL: interleukin
LPS: lipopolysaccharide
MOS: McGill Oximetry Score
NFκB: nuclear factor kappa B
OSA: obstructive sleep apnea
PBMC: peripheral blood mononuclear cells
TNFα: tumor necrosis factor alpha
